# Efficacy and safety of GLP-1 receptor agonists in patients with metabolic dysfunction-associated steatotic liver disease (MASLD) and metabolic dysfunction-associated steatohepatitis (MASH): A systematic review and meta-analysis

**DOI:** 10.1097/MD.0000000000051306

**Published:** 2026-10-23

**Authors:** Mohammed Alfawaz, Dareen Bajamaan, Lama Alghamdi, Yara Alorfi, Saud Khared, Nada Ameen, Rahaf Alamri, Mohammed Abuladel, Rayan Alshehri, Shaima Altowairqi

**Affiliations:** a Department of Internal Medicine, Gastroenterology Consultant, College of Medicine, University of Jeddah, Jeddah, Saudi Arabia; b Medical Intern, College of Medicine, University of Jeddah, Jeddah, Saudi Arabia; c Internal Medicine Resident, Department of Internal Medicine, King Faisal Specialist Hospital and Research Centre, Jeddah, Saudi Arabia.

**Keywords:** GLP-1 receptor agonists, liraglutide, liver fat, meta-analysis, metabolic dysfunction-associated steatohepatitis, metabolic dysfunction-associated steatotic liver disease, semaglutide

## Abstract

**Background:**

Metabolic dysfunction-associated steatotic liver disease (MASLD) and metabolic dysfunction-associated steatohepatitis (MASH) are increasingly prevalent liver diseases closely linked to obesity, type 2 diabetes mellitus, and cardiometabolic risk. Glucagon-like peptide-1 receptor agonists (GLP-1 RAs) may improve hepatic and metabolic outcomes through weight reduction, improved glycemic control, and reductions in hepatic steatosis. This systematic review and meta-analysis aimed to evaluate the efficacy and safety of GLP-1 RAs in adults with MASLD or MASH.

**Methods:**

A systematic review and meta-analysis was conducted in accordance with PRISMA 2020 guidelines. PubMed, Web of Science, Scopus, Google Scholar, and the Cochrane Central Register of Controlled Trials were searched for English-language studies published between January 2019 and December 2025. Eligible studies included adults with MASLD or MASH treated with GLP-1 RAs and reporting hepatic, metabolic, or safety outcomes. Pooled estimates were calculated using random-effects models.

**Results:**

Nine studies involving 795 participants were included. GLP-1 RA therapy was associated with significant reductions in liver fat content (mean difference [MD]: −3.17; 95% CI: −4.06 to −2.29), body weight (MD: −4.68; 95% CI: −6.88 to −2.47), alanine aminotransferase (MD: −15.17; 95% CI: −21.29 to −9.05), aspartate aminotransferase (MD: −9.89; 95% CI: −13.65 to −6.13), and HbA1c (MD: −0.78; 95% CI: −1.07 to −0.49). Adverse events were more frequent with GLP-1 RAs, particularly nausea and vomiting, while serious adverse events did not differ significantly between groups.

**Conclusion:**

GLP-1 RAs are associated with meaningful improvements in hepatic steatosis, liver enzymes, body weight, and glycemic control in patients with MASLD or MASH. These findings support their potential role, particularly in patients with coexisting obesity or type 2 diabetes. However, further long-term, adequately powered trials are needed to clarify their effects on fibrosis regression, histological outcomes, and liver-related clinical events.

## 1. Introduction

Metabolic dysfunction–associated steatotic liver disease (MASLD), previously known as non fatty liver disease (NAFLD), is a common chronic liver disease characterized by hepatic fat accumulation in the absence of secondary causes.^[1]^ It is strongly associated with metabolic risk factors, particularly obesity and type 2 diabetes mellitus, and affects approximately 25 to 38% of the global adult population, with higher prevalence in high-risk groups.^[1,2]^ The disease encompasses a spectrum ranging from simple steatosis to metabolic dysfunction–associated steatohepatitis (MASH) which previously was known as Nonalcoholic steatohepatitis (NASH), an inflammatory phenotype associated with hepatocellular injury.^[3]^

Clinically, MASLD is associated with progressive hepatic fibrosis, which is the strongest predictor of liver-related outcomes, including cirrhosis and hepatocellular carcinoma.^[3–5]^ In parallel, MASLD contributes to an increasing global burden of hepatocellular carcinoma and is also strongly linked to cardiovascular morbidity and mortality, which remains the leading cause of death in this population. Together, these complications highlight the systemic and progressive nature of the disease.^[4,5]^

Despite its high prevalence, effective pharmacological options remain limited. Current management is primarily based on lifestyle modification, including dietary intervention, physical activity, and weight reduction; however, long-term adherence is often poor and sustained histological improvement is difficult to achieve.^[6]^ Pharmacological agents such as Pioglitazone and vitamin E have shown benefit in selected patients but are limited by safety concerns and inconsistent antifibrotic effects.^[7]^ More recently, Resmetirom (MGL-3196), a thyroid hormone receptor-β agonist, has demonstrated improvement in hepatic fat content and histological features of MASH in patients with fibrosis (F2–F3), although long-term clinical outcomes remain uncertain.^[8]^ Overall, there remains a significant unmet need for therapies targeting both metabolic dysfunction and fibrotic progression.^[9]^

In this context, GLP-1 receptor agonists have emerged as promising therapeutic agents due to their metabolic and potential hepatic benefits.^[10,11]^ These agents enhance glucose-dependent insulin secretion, suppress glucagon release, and improve metabolic homeostasis, leading to clinically meaningful weight loss, a key driver of improvement in hepatic steatosis and inflammation.^[10]^ Randomized controlled trials have shown that semaglutide increases MASH resolution rates compared with placebo, although fibrosis improvement remains limited.^[11]^

Current consensus further supports MASLD/MASH as a metabolic-driven disease with limited antifibrotic therapies, reinforcing the need for novel treatment strategies.^[9]^ Collectively, these findings support GLP-1 receptor agonists as promising candidates targeting both metabolic and hepatic disease pathways, although long-term outcomes remain under investigation.^[9–11]^

This systematic review aims to critically evaluate the efficacy and safety of glucagon-like peptide-1 receptor agonists (GLP-1 RAs) in adults with metabolic dysfunction-associated steatotic liver disease (MASLD) and metabolic dysfunction-associated steatohepatitis (MASH), compared with standard care or placebo. The review will assess their effects on hepatic steatosis, histological outcomes (inflammation and fibrosis), biochemical markers of liver injury, metabolic parameters, and clinically relevant disease progression

## 2. Materials and methods

### 2.1. Study design

This systematic review and meta-analysis was conducted in accordance with the Preferred Reporting Items for Systematic Reviews and Meta-Analyses 2020 statement (PRISMA 2020). The review protocol was prospectively registered in the International Prospective Register of Systematic Reviews (PROSPERO) under registration number CRD420261298457.

### 2.2. Eligibility criteria

Studies were selected according to the Population, Intervention, Comparison, and Outcomes (PICO) framework. The population of interest included adults aged ≥ 18 years with metabolic dysfunction-associated steatotic liver disease (MASLD) or metabolic dysfunction-associated steatohepatitis (MASH). The intervention of interest was treatment with glucagon-like peptide-1 receptor agonists (GLP-1 RAs), including semaglutide, liraglutide, dulaglutide, or other GLP-1 RA-based therapies. Eligible comparators included placebo, standard care, lifestyle intervention, no pharmacological intervention, or active treatment arms, where applicable. The primary outcomes were changes in liver fat content, liver enzyme levels, including alanine aminotransferase (ALT) and aspartate aminotransferase (AST), and histological liver outcomes when reported. Secondary outcomes included changes in body weight, body mass index (BMI), glycated hemoglobin (HbA1c), insulin resistance assessed using the homeostatic model assessment of insulin resistance (HOMA-IR), and safety outcomes, including overall adverse events, serious adverse events, nausea, vomiting, and diarrhea. Eligible study designs included randomized controlled trials and non-randomized interventional studies, including single-arm trials. Only full-text articles published in English between January 2019 and December 2025 were included. Studies were excluded if they included pediatric populations, did not report liver-related outcomes, focused exclusively on weight loss or glycemic control without hepatic assessment, were not original human research articles, or were published in languages other than English.

### 2.3. Information sources and search strategy

A comprehensive literature search was conducted using PubMed, Web of Science, Scopus, Google Scholar, and the Cochrane Central Register of Controlled Trials. The search covered studies published between January 2019 and December 2025. The search strategy was developed using combinations of Medical Subject Headings and free-text terms related to GLP-1 receptor agonists and MASLD/MASH. The following search terms and Boolean operators were used: (“GLP-1 receptor agonists” OR semaglutide OR liraglutide OR dulaglutide) AND (“metabolic dysfunction-associated steatotic liver disease” OR MASLD OR “metabolic dysfunction-associated steatohepatitis” OR MASH OR “nonalcoholic fatty liver disease” OR NAFLD OR “nonalcoholic steatohepatitis” OR NASH) AND (“liver fat” OR MRI-PDFF OR “liver enzymes” OR ALT OR AST OR fibrosis OR steatosis). The search strategy was adapted as needed for each database. Retrieved records were screened after duplicate removal. No backward or forward citation tracking was performed (Fig. 1**).**

**Figure 1.**
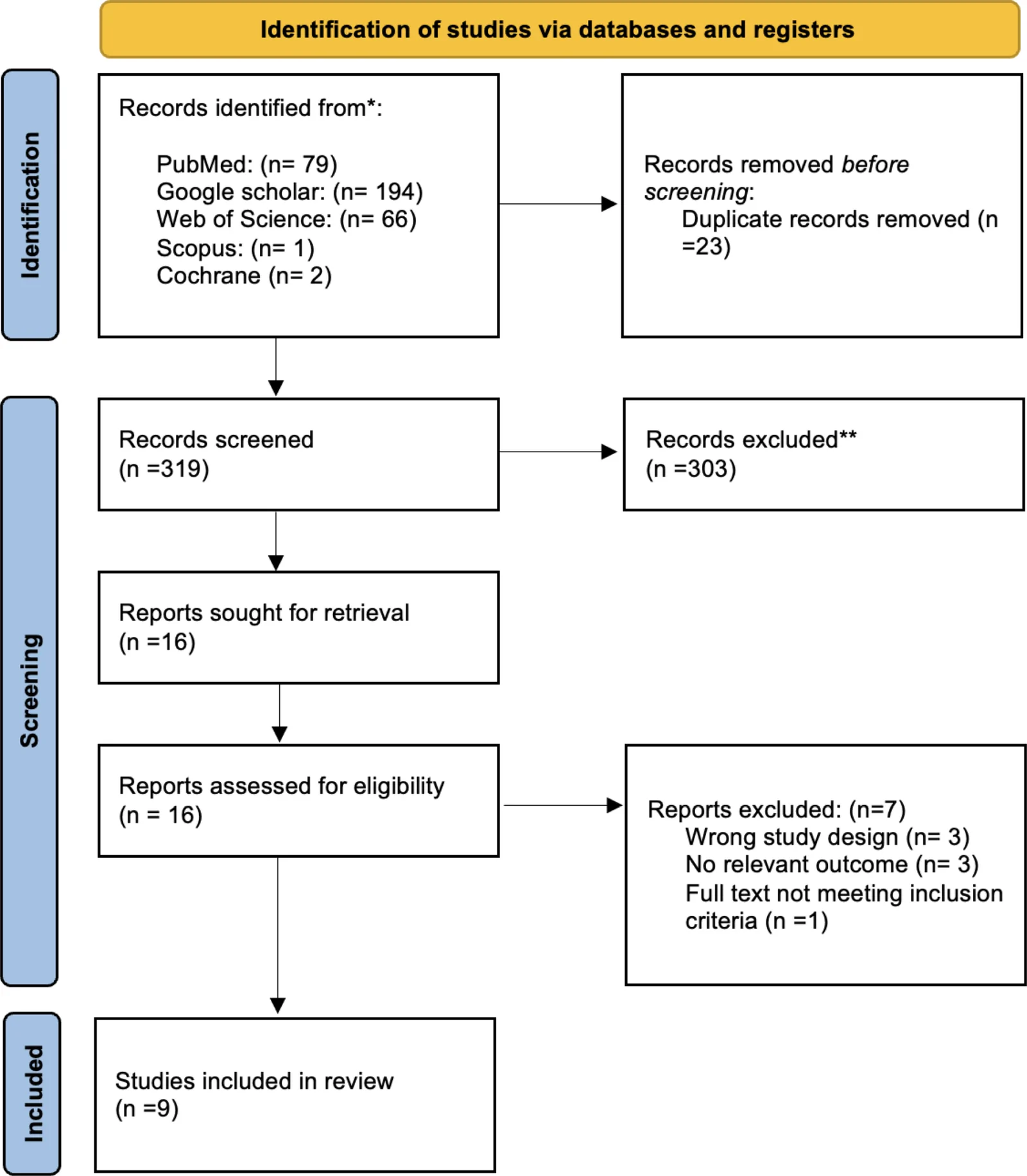
The PRISMA chart of the included studies.

### 2.4. Study selection and data extraction

Study selection was performed in 2 stages. First, titles and abstracts of retrieved records were screened for relevance to the review question. Second, the full texts of potentially eligible articles were retrieved and assessed against the predefined inclusion and exclusion criteria. Disagreements between reviewers were resolved through discussion and consensus. Data were extracted independently by 2 reviewers using a pre data extraction form. Extracted data included study characteristics, such as author name, publication year, country, study design, sample size, comparator, and follow-up duration. Participant characteristics included age, sex, baseline body mass index, diagnostic criteria for MASLD or MASH, and method of liver disease assessment. Intervention details included the type of GLP-1 receptor agonist, dose, route of administration, and treatment duration. Comparator details included placebo, standard care, lifestyle intervention, no intervention, or active comparator where applicable. Outcome data were extracted for liver fat content, liver enzymes, histological outcomes, body weight, BMI, HbA1c, HOMA-IR, and adverse events. For randomized controlled trials with sufficient comparative data, outcomes were considered for quantitative pooling. The single-arm interventional study was summarized narratively when comparative pooling was not appropriate. When studies included multiple eligible treatment arms, relevant arms were handled carefully to avoid double-counting participants.

### 2.5. Quality assessment

The methodological quality and risk of bias of included studies were assessed according to study design. Randomized controlled trials were evaluated using the Cochrane Risk of Bias 2 tool (RoB 2), which assesses bias arising from the randomization process, deviations from intended interventions, missing outcome data, outcome measurement, and selection of reported results. The single-arm interventional study was assessed separately using the Risk Of Bias In Non-randomized Studies of Interventions tool (ROBINS-I), with consideration of the inherent limitations caused by the absence of a concurrent control group. Domains assessed included bias due to confounding, participant selection, intervention classification, deviations from intended interventions, missing data, outcome measurement, and selection of reported results. Risk-of-bias assessments were performed independently by 2 reviewers, and disagreements were resolved through consensus. Overall judgments were categorized according to the guidance of each tool. Risk-of-bias results were presented graphically and summarized narratively (Fig. 2).

**Figure 2.**
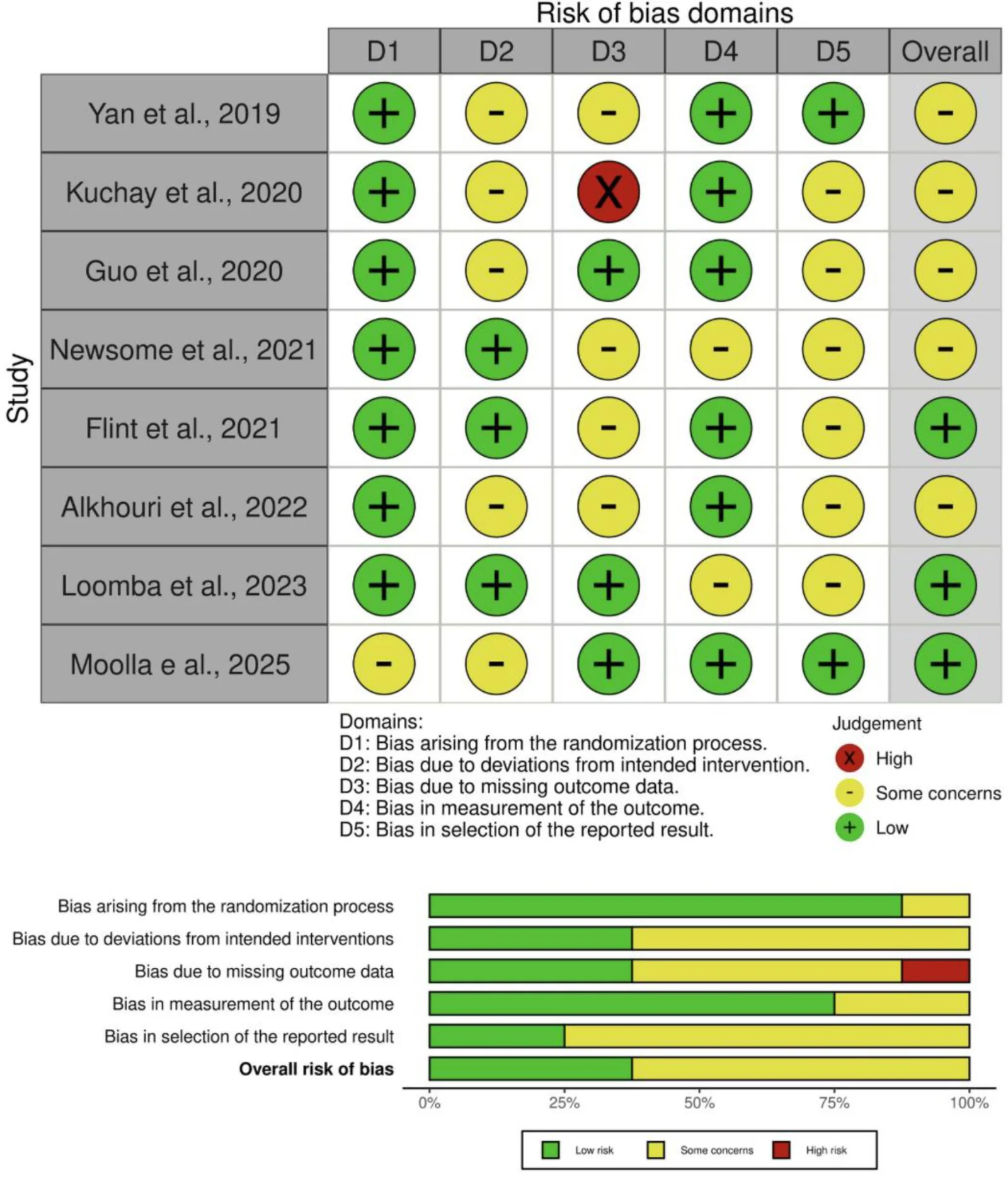
Risk of Bias assessment of the included studies.

### 2.6. Certainty of evidence

The certainty of evidence for each quantitatively synthesized outcome was assessed using the Grading of Recommendations Assessment, Development and Evaluation (GRADE) approach. Randomized controlled trial evidence was initially considered high certainty and was subsequently evaluated for potential downgrading across the domains of risk of bias, inconsistency, indirectness, imprecision, and publication bias. Certainty of evidence was categorized as high, moderate, low, or very low. Publication bias could not be formally evaluated because fewer than ten studies contributed to each pooled outcome.

### 2.7. Statistical analysis

Meta-analyses were conducted using Review Manager software (RevMan) version 5.4.1. Outcomes reported by at least 2 studies with sufficient quantitative and comparative data were included in the quantitative synthesis. Continuous outcomes, including liver fat content, body weight, ALT, AST, and HbA1c, were summarized using mean differences (MDs) with 95% confidence intervals (CIs). Dichotomous safety outcomes, including overall adverse events, serious adverse events, nausea, vomiting, and diarrhea, were summarized using risk ratios (RRs) with 95% CIs. Pooled estimates were calculated using a random-effects model to account for expected clinical and methodological heterogeneity across studies. Statistical heterogeneity was assessed using the Chi-square test, Tau^2^, and the I^2^ statistic. I^2^ values were interpreted according to their magnitude and clinical context, with higher values indicating greater between-study heterogeneity. For outcomes demonstrating substantial heterogeneity (I^2^ ≥ 75%), leave-one-out sensitivity analyses were performed by sequentially excluding each individual study and recalculating the pooled effect estimate and heterogeneity statistics to assess the robustness of the findings and identify potentially influential studies. Meta-regression was not performed because the small number of studies contributing to the relevant pooled outcome was considered insufficient for reliable covariate analysis. A *P*-value of < .05 was considered statistically significant. The single-arm study was not included in comparative meta-analyses when no control group was available and was instead described narratively. Where quantitative pooling was not appropriate because of differences in study design, outcome definition, comparator type, or reporting format, findings were synthesized narratively. Publication bias was not formally assessed because fewer than ten studies were available for each pooled outcome.

### 2.8. Ethical approval

Ethical approval and institutional review board approval were not required for this systematic review and meta-analysis because the study used data from previously published studies and did not involve direct recruitment of human participants or collection of identifiable individual-level patient data.

## 3. Results

### 3.1. Characteristics of the included studies

Nine studies were included in the systematic review and meta-analysis, comprising a total of 795 participants.^[12–19]^ Across the included studies, sample sizes ranged from 30 to 320 participants. Most studies were randomized controlled trials, with one single-arm trial. The studies were conducted across multiple countries, including the USA, China, India, Denmark, the UK, and multinational settings. Participants were allocated into intervention and control arms in most studies, with comparators including placebo, standard diabetes care, lifestyle intervention, or active treatments. The liver disease types included Metabolic dysfunction–associated steatotic liver disease (MASLD) and metabolic dysfunction–associated steatohepatitis (MASH), assessed using liver biopsy, MRI-based techniques, or imaging and biomarker-based methods. Interventions primarily involved GLP-1 receptor agonists, most commonly semaglutide, administered subcutaneously, with treatment durations ranging from 12 to 72 weeks (Table 1).

**Table 1:** Characteristics of included studies.

Study	Country	Study Design	Total Sample Size	Groups (Intervention/ Control)	Comparator	Liver Disease Type	Liver Disease Assessment	Intervention	Dose/ Route	Treatment Duration
**Yan et al** ^[12]^	China	Randomized trial	75	Three intervention arms	Active treatment arms	MASLD	Imaging	GLP-1-based therapy	Subcutaneous	12–24 weeks
**Kuchay et al** ^[13]^	India	Randomized controlled trial	30	Intervention vs standard care	Standard diabetes care	MASLD	MRI-PDFF	Semaglutide	Subcutaneous	24 weeks
**Guo et al** ^[14]^	China	Randomized controlled trial	66	Intervention vs placebo	Placebo	MASLD	Imaging assessment	Liraglutide	Subcutaneous	24 weeks
**Newsome et al** ^[11]^	Multinational	Randomized controlled trial	320	Intervention vs placebo	Placebo	MASH	Liver biopsy	Semaglutide	Subcutaneous	72 weeks
**Flint et al** ^[15]^	Denmark	Randomized controlled trial	72	Intervention vs placebo	Placebo	MASLD	MRI-based assessment	Semaglutide	Subcutaneous	48 weeks
**Alkhouri et al** ^[16]^	USA	Randomized clinical trial	67	Combination therapy vs monotherapy	Semaglutide monotherapy	MASH	Liver biopsy	Combination therapy	Subcutaneous	48 weeks
**Loomba et al** ^[17]^	USA	Randomized controlled trial	71	Intervention vs placebo	Placebo	MASH	MRI-PDFF	Semaglutide	Subcutaneous	48 weeks
**Moolla et al** ^[18]^	UK	Randomized controlled trial	64	Intervention vs lifestyle	Lifestyle intervention	MASLD	MRI-PDFF	Semaglutide	Subcutaneous	24 weeks
**Liu et al** ^[19]^	China	Single-arm trial	30	Intervention only	None	MASLD	Imaging/ biomarkers	Semaglutide	Subcutaneous	24 weeks

GLP-1 = glucagon-like peptide-1, MASH = metabolic dysfunction-associated steatohepatitis, MASLD = metabolic dysfunction-associated steatotic liver disease, MRI = magnetic resonance imaging, MRI-PDFF = magnetic resonance imaging-proton density fat fraction, UK = United Kingdom, USA = United States of America.

Baseline participant characteristics varied across studies. The mean age of participants ranged from 39.6 to 60.5 years. The proportion of male participants varied across studies, with reported distributions ranging from 17/7 to 47/20 in intervention groups and similar variability in control groups. Mean body mass index (BMI) ranged from approximately 28.3 to 36.4 kg/m^2^ across studies (Table 2).

**Table 2:** Baseline participant characteristics of included studies.

Study	Mean Age (years)	Sex (Male/Female)	Mean BMI (kg/m^2^)
**Yan et al** ^[12]^	Liraglutide: 43.1 ± 9.7; Sitagliptin: 45.7 ± 9.2; Insulin glargine: 45.6 ± 7.6	Liraglutide: 17/7; Sitagliptin: 21/6; Insulin glargine: 14/10	Liraglutide: 30.1 ± 3.3; Sitagliptin: 29.7 ± 2.8; Glargine: 29.6 ± 3.5
**Kuchay et al** ^[13]^	Dulaglutide: 46.6 ± 9.1; Control: 48.1 ± 8.9	Dulaglutide: 23/9; Control: 22/10	Dulaglutide: 29.6 ± 3.6; Control: 29.9 ± 3.9
**Guo et al** ^[14]^	Glargine: 52.0 ± 8.7; Liraglutide: 53.1 ± 6.3; Placebo: 52.6 ± 3.9	Glargine: 18/12; Liraglutide: 16/15; Placebo: 20/10	Glargine: 28.3 ± 3.8; Liraglutide: 29.2 ± 4.2; Placebo: 28.6 ± 3.7
**Newsome et al** ^[11]^	Semaglutide 0.1 mg: 55.2 ± 10.9; 0.2 mg: 58.1 ± 9.9; 0.4 mg: 54.3 ± 10.2; Placebo: 52.4 ± 10.8	0.1 mg: 29/51; 0.2 mg: 26/52; 0.4 mg: 35/47; Placebo: 36/44	0.1 mg: 36.1 ± 6.4; 0.2 mg: 35.6 ± 6.1; 0.4 mg: 35.2 ± 6.6; Placebo: 36.1 ± 6.6
**Flint et al** ^[15]^	Semaglutide: 59.5 ± 10.1; Placebo: 60.5 ± 8.5	47/ 20	94% BMI ≥ 30
**Alkhouri et al** ^[16]^	Median 54 (48–61)	34/ 74	Median range 33.2–39.5
**Loomba et al** ^[17]^	59.5 ± 8.0	22/ 49	34.9 ± 5.9
**Moolla et al** ^[18]^	Lifestyle: 48 ± 4; Liraglutide: 48 ± 4	Lifestyle: 50% male; Liraglutide: 53.3% male	Lifestyle: 36.4 ± 1.5; Liraglutide: 35.7 ± 1.7
**Liu et al** ^[19]^	39.55 ± 11.58	32/ 6	Median 29.35 (IQR 25.56–47.97)

BMI = body mass index, IQR = interquartile range, kg/m^2^ = kilograms per square meter, mg = milligram.

### 3.2. Meta-analysis

#### 3.2.1. Change in liver fat content

Three studies reported a change in liver fat content.^[12–14]^ Pooled analysis showed that the intervention group had a greater reduction in liver fat content than the control group, with a mean difference of −3.17 (95% CI: −4.06 to −2.29). The overall effect was statistically significant (Z = 7.00, *P* < .00001). Heterogeneity was not observed across the included studies (Tau^2^ = 0.00; Chi^2^ = 0.84, df = 2, *P* = .66; I^2^ = 0%) (Fig. 3).

**Figure 3.**
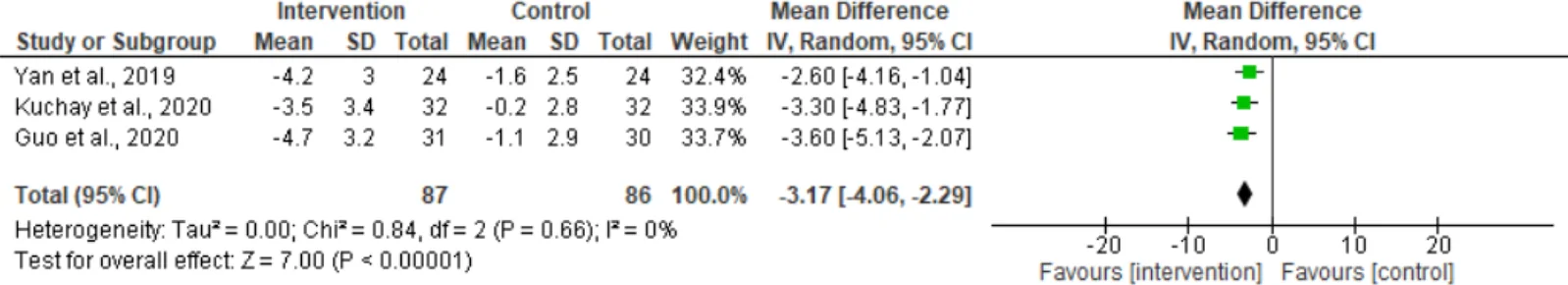
Meta-analysis of the change in liver fat content.

#### 3.2.2. Change in body weight

Four studies reported a change in body weight.^[12,14,17,18]^ The pooled analysis demonstrated that the intervention was associated with a greater reduction in body weight compared with the control group, with a mean difference of − 4.68 (95% CI: −6.88 to −2.47). This effect was statistically significant (Z = 4.16, *P* < .0001). However, substantial heterogeneity was observed among the included studies (Tau^2^ = 4.09; Chi^2^ = 17.10, df = 3, *P* = .0007; I^2^ = 82%) (Fig. 4). Leave-one-out sensitivity analysis showed that the pooled effect remained statistically significant after sequentially excluding each study, with pooled mean differences ranging from − 3.64 to − 5.48. Heterogeneity remained substantial in most analyses (I^2^ = 80–87%); however, excluding Loomba et al reduced I^2^ to 64%, suggesting this study contributed to but did not fully explain the observed between-study heterogeneity. Overall, the direction and statistical significance of the pooled body-weight effect were robust to the exclusion of individual studies.

**Figure 4.**
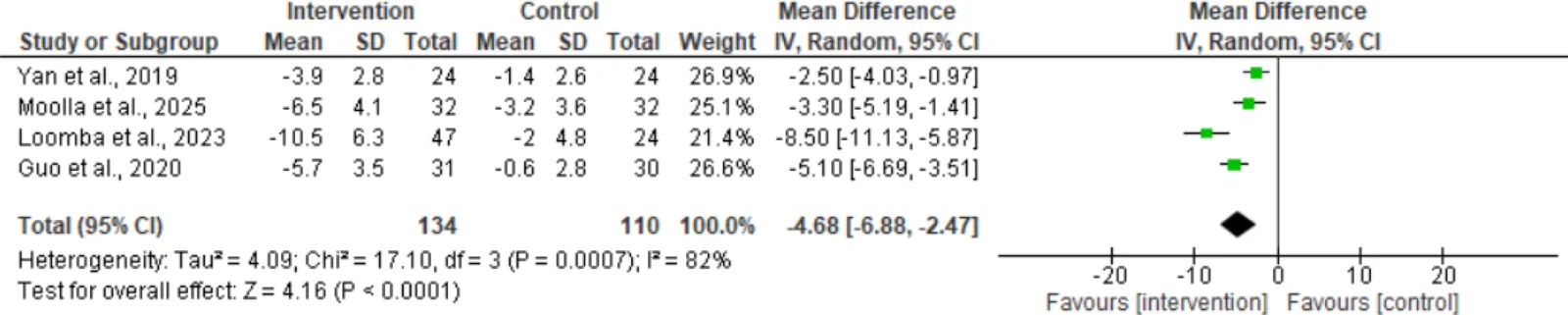
Meta-analysis of the change in body weight.

#### 3.2.3. Change in ALT, AST, and HbA1C

Two studies reported a change in ALT, 2 studies reported a change in AST, and 2 studies reported a change in HbA1c.^[12–14]^ The pooled results showed that the intervention was associated with greater reductions in ALT (mean difference: −15.17; 95% CI: −21.29 to −9.05), AST (mean difference: −9.89; 95% CI: −13.65 to −6.13), and HbA1c (mean difference: −0.78; 95% CI: −1.07 to −0.49) compared to the control group. All effects were statistically significant (*P* < .00001 for each outcome). No heterogeneity was observed for ALT and AST (I^2^ = 0% for both), while low-to-moderate heterogeneity was noted for HbA1c (I^2^ = 38%) (Fig. 5).

**Figure 5.**
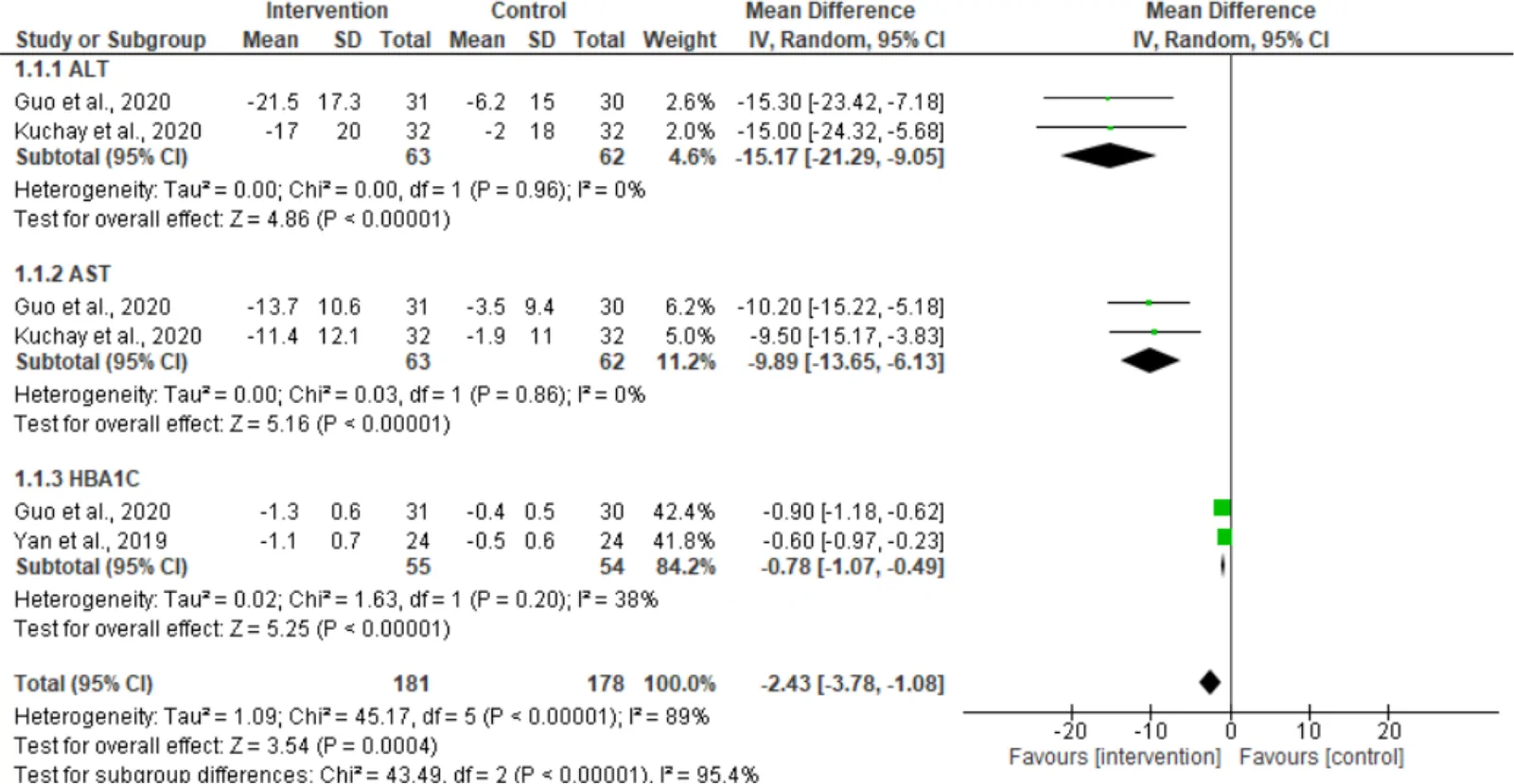
Meta-analysis of the change in ALT, AST, and HbA1C.

#### 3.2.4. All adverse events

Three studies reported all adverse events.^[11,15,17]^ The pooled analysis indicated that adverse events were more frequent in the intervention group compared to the control group, with a risk ratio of 1.13 (95% CI: 1.02 to 1.25). This difference was statistically significant (Z = 2.31, *P* = .02). No heterogeneity was observed among the included studies (Tau^2^ = 0.00; Chi^2^ = 0.83, df = 2, *P* = .66; I^2^ = 0%) (Fig. 6).

**Figure 6.**
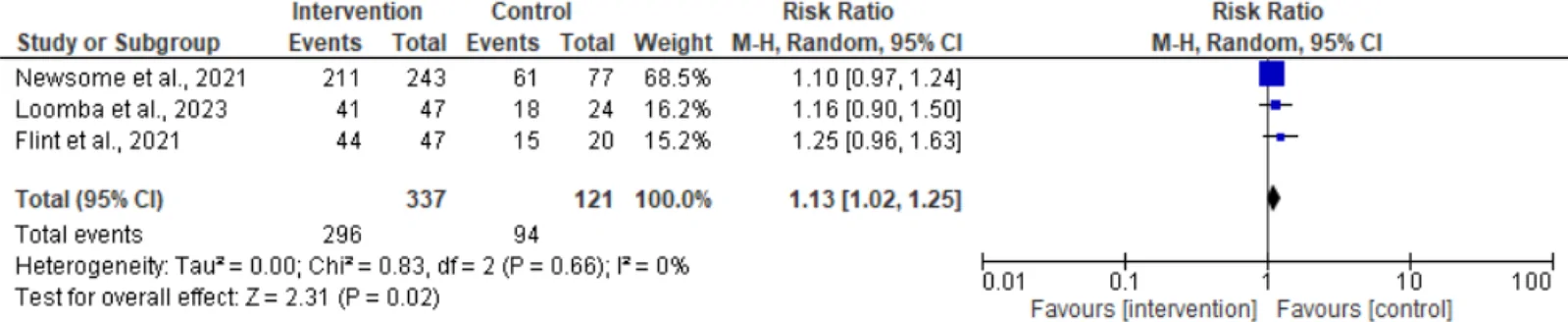
Meta-analysis of the risk of all adverse events.

#### 3.2.5. Serious adverse events

Three studies reported serious adverse events.^[11,15,17]^ The pooled analysis showed no significant difference between the intervention and control groups, with a risk ratio of 1.37 (95% CI: 0.52 to 3.62). This finding was not statistically significant (Z = 0.64, *P* = .52). There was no evidence of heterogeneity among the included studies (Tau^2^ = 0.00; Chi^2^ = 0.29, df = 2, *P* = .86; I^2^ = 0%) (Fig. 7).

**Figure 7.**
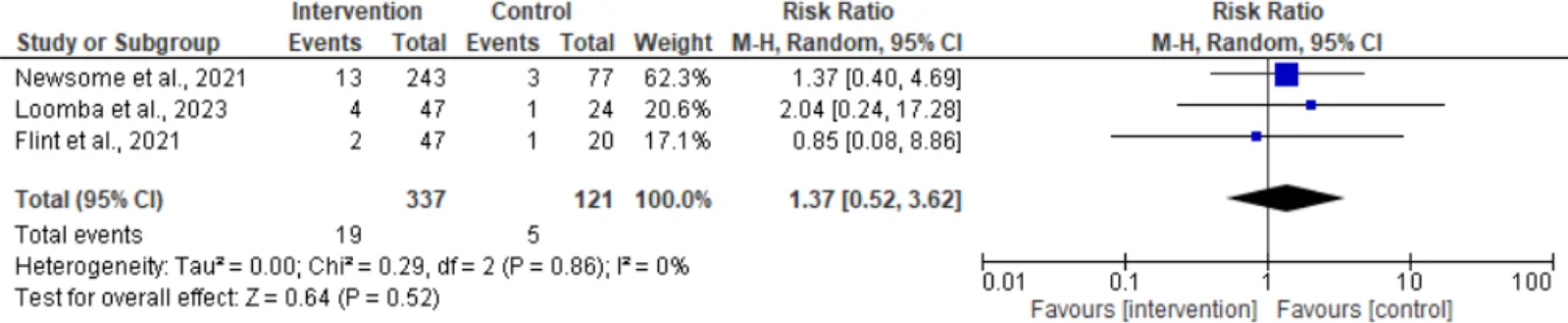
Meta-analysis of the risk of serious adverse events.

#### 3.2.6. Nausea

Three studies reported nausea.^[11,14,15]^ The pooled analysis demonstrated that nausea occurred more frequently in the intervention group compared to the control group, with a risk ratio of 1.96 (95% CI: 1.34 to 2.86). This difference was statistically significant (Z = 3.49, *P* = .0005). No heterogeneity was observed across the included studies (Tau^2^ = 0.00; Chi^2^ = 1.37, df = 2, *P* = .51; I^2^ = 0%) (Fig. 8).

**Figure 8.**
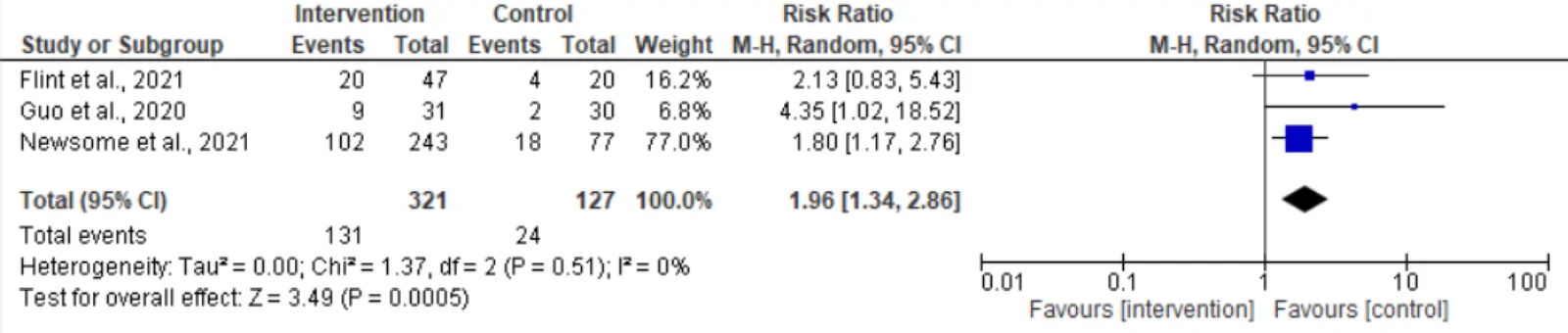
Meta-analysis of the risk of nausea.

#### 3.2.7. Vomiting

Three studies reported vomiting.^[11,14,15]^ The pooled analysis showed that vomiting was more frequent in the intervention group compared to the control group, with a risk ratio of 2.34 (95% CI: 1.20 to 4.56). This difference reached statistical significance (Z = 2.49, *P* = .01). There was no evidence of heterogeneity among the included studies (Tau^2^ = 0.00; Chi^2^ = 0.44, df = 2, *P* = .80; I^2^ = 0%) (Fig. 9).

**Figure 9.**
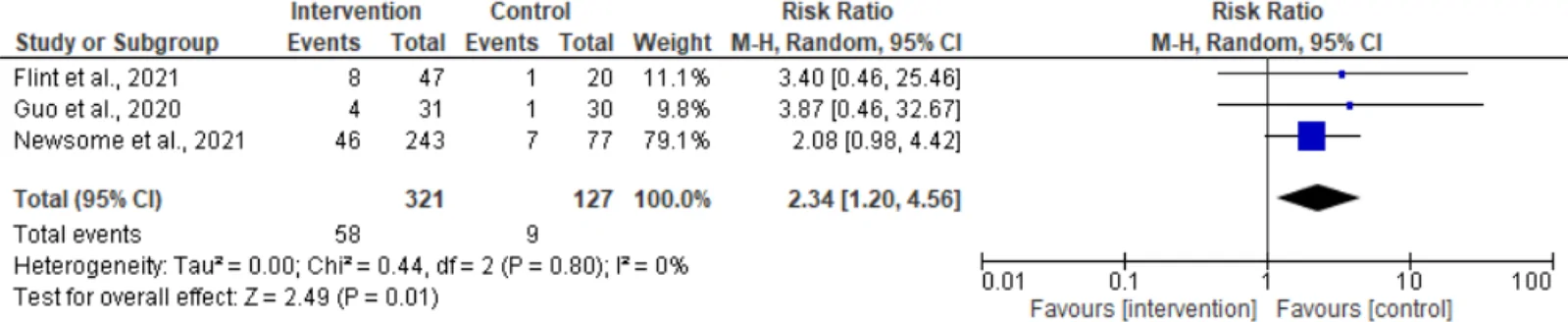
Meta-analysis of the risk of vomiting.

#### 3.2.8. Diarrhea

Three studies reported diarrhea.^[11,14,15]^ The pooled analysis showed a higher occurrence of diarrhea in the intervention group compared to the control group, with a risk ratio of 1.77 (95% CI: 0.93 to 3.38). However, this difference was not statistically significant (Z = 1.74, *P* = .08). No heterogeneity was detected among the included studies (Tau^2^ = 0.00; Chi^2^ = 1.22, df = 2, *P* = .54; I^2^ = 0%) (Fig. 10).

**Figure 10.**
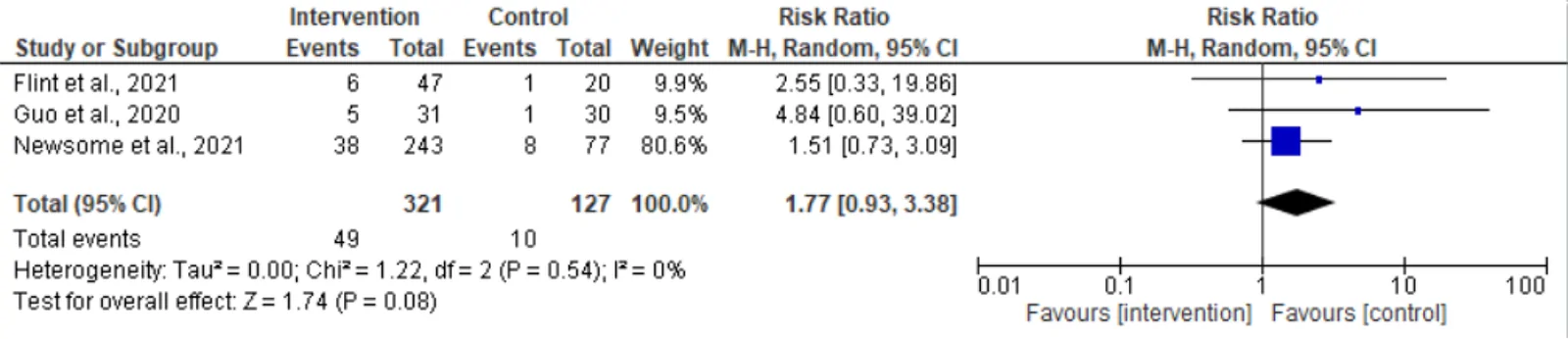
Meta-analysis of the risk of diarrhea.

### 3.3. Certainty of evidence

The certainty of evidence ranged from very low to moderate across the assessed outcomes. Certainty was limited primarily by risk-of-bias concerns, small numbers of contributing studies, imprecision, and, for body weight, substantial inconsistency. Table 3 summarizes the GRADE assessments for all quantitatively synthesized outcomes (Table 3).

**Table 3:** Summary of findings and GRADE certainty of evidence.

Outcome	Studies/ participants	Pooled effect (95% CI)	Inconsistency	Main certainty concerns	GRADE certainty
**Liver fat content**	3 studies/ 173	MD −3.17 (−4.06 to −2.29)	I^2^ = 0%	Risk of bias; limited evidence base	**Low ⨁⨁◯◯**
**Body weight**	4 studies/ 244	MD −4.68 (−6.88 to −2.47)	I^2^ = 82%	Risk of bias; serious inconsistency	**Low ⨁⨁◯◯**
**ALT**	2 studies/ 125	MD −15.17 (−21.29 to −9.05)	I^2^ = 0%	Risk of bias; imprecision due to small evidence base	**Low ⨁⨁◯◯**
**AST**	2 studies/ 125	MD −9.89 (−13.65 to −6.13)	I^2^ = 0%	Risk of bias; imprecision due to small evidence base	**Low ⨁⨁◯◯**
**HbA1c**	2 studies/ 109	MD −0.78 (−1.07 to −0.49)	I^2^ = 38%	Risk of bias; imprecision due to small evidence base	**Low ⨁⨁◯◯**
**All adverse events**	3 studies/ 458	RR 1.13 (1.02 to 1.25)	I^2^ = 0%	Risk of bias	**Moderate ⨁⨁⨁◯**
**Serious adverse events**	3 studies/ 458	RR 1.37 (0.52 to 3.62)	I^2^ = 0%	Risk of bias; very serious imprecision	**Very low ⨁◯◯◯**
**Nausea**	3 studies/ 448	RR 1.96 (1.34 to 2.86)	I^2^ = 0%	Risk of bias	**Moderate ⨁⨁⨁◯**
**Vomiting**	3 studies/ 448	RR 2.34 (1.20 to 4.56)	I^2^ = 0%	Risk of bias; imprecision	**Low ⨁⨁◯◯**
**Diarrhea**	3 studies/ 448	RR 1.77 (0.93 to 3.38)	I^2^ = 0%	Risk of bias; imprecision	**Low ⨁⨁◯◯**

## 4. Discussion

The pooled reduction in liver fat content supports a consistent effect of GLP-1–based therapy on hepatic steatosis across the contributing trials. This finding aligns closely with the direction and magnitude reported in an updated meta-analysis of phase 2 RCTs by Mantovani and colleagues,^[20]^ which found a significant reduction in MRI-assessed absolute liver fat percentage (WMD= −3.9) over a median treatment duration of 26 weeks. Convergence between these pooled estimates reinforces the plausibility that GLP-1 RA–mediated weight loss and metabolic improvement translate into measurable decreases in intrahepatic lipid, particularly when quantified using MRI-based techniques that are sensitive to change over relatively short intervals.

Our results extend and complement prior biopsy-focused MASLD/MASH trials by demonstrating consistent improvements in steatosis and metabolic markers in pooled analyses dominated by imaging-defined endpoints. Notably, the phase 2 biopsy-confirmed MASH trial of subcutaneous semaglutide demonstrated dose-responsive histologic efficacy for MASH resolution without worsening fibrosis (59% with 0.4 mg vs 17% placebo), establishing proof-of-concept that GLP-1 RA therapy can impact disease activity beyond steatosis alone, although fibrosis improvement remained more challenging.^[11]^ Earlier, the LEAN trial (liraglutide 1.8 mg daily) also reported higher MASH resolution (39% vs 9% placebo; RR 4.3) and fewer participants with fibrosis progression, supporting class-level potential while underscoring variability by molecule, dose, and stage.^[21]^ In MASLD populations assessed by MRI-based methods, semaglutide reduced liver steatosis but not liver stiffness in a randomized placebo-controlled trial,^[15]^ consistent with the broader pattern that steatosis and transaminases may improve over months while fibrosis-related endpoints often require longer exposure, larger samples, and/or combination strategies. In more advanced disease, semaglutide 2.4 mg weekly in MASH-related compensated cirrhosis provides an important boundary condition for expectations in late-stage populations, where short- to medium-term histologic reversal is particularly difficult to demonstrate.^[17]^

Positioning GLP-1 RAs within the broader MASLD/MASH therapeutic landscape highlights a recurring trade-off: agents that drive weight loss and metabolic improvement tend to show robust effects on steatosis and disease activity, whereas direct antifibrotic effects remain less consistently achieved. For example, in PIVENS (nondiabetic MASH), vitamin E improved histologic outcomes versus placebo (43% vs 19%), while pioglitazone did not meet the same threshold for significance in that trial (34% vs 19%), illustrating both the potential and limitations of “activity-targeted” approaches.^[7]^ In contrast, FXR agonism with obeticholic acid has shown more direct antifibrotic signal: REGENERATE interim analysis demonstrated fibrosis improvement (≥1 stage) with no worsening of MASH in 23% with obeticholic acid 25 mg versus 12% with placebo, although the MASH-resolution co-primary endpoint was not met at month 18.^[22]^ Earlier, the FLINT trial similarly provided evidence that FXR agonism can improve histologic features in noncirrhotic MASH.^[23]^

SGLT2 inhibitors offer a useful metabolic comparator given their modest weight loss and glycemic benefits. In the E-LIFT RCT, empagliflozin added to standard diabetes care reduced MRI-PDFF liver fat relative to control (between-group difference −4.0%) and improved ALT, supporting a steatosis-lowering effect that is broadly comparable in magnitude to GLP-1 RA pooled liver fat reductions, though through distinct mechanisms and typically with smaller weight loss.^[24]^ A meta-analysis of randomized clinical trials on type 2 diabetes patients with MASLD also supports improvements in liver-related outcomes with SGLT2 inhibitors, reinforcing that multiple metabolic drug classes can deliver measurable hepatic benefits but may differ in tolerability profiles, weight effects, and evidence strength for histologic endpoints.^[25]^ Finally, rational combination therapy is an active development strategy. A randomized open-label phase 2 trial combining semaglutide with cilofexor and/or firsocostat reported comparable weight loss across arms but larger MRI-PDFF improvements and biochemical changes with combination regimens than semaglutide alone in exploratory analyses, hypothesis-generating data that may be relevant for future fibrosis-focused programs.^[16]^

From a clinical decision-making perspective, these data support GLP-1 RAs as a therapy that can deliver concurrent improvements in hepatic steatosis, liver enzymes, body weight, and glycemic control – domains that align with major drivers of MASLD progression and cardiometabolic risk. The strongest near-term rationale is therefore in patients with MASLD/MASH who also meet established indications for GLP-1 RA therapy (type 2 diabetes and/or obesity), where the hepatic effects may be considered an added-value outcome rather than the sole treatment objective. Evidence linking ≥ 10% weight loss to high rates of MASH resolution and meaningful fibrosis regression underscores why therapies that achieve durable weight reduction can be clinically relevant for liver disease trajectories, even when antifibrotic effects are not immediately demonstrable in short trials.^[26]^ In practice, tolerability-driven discontinuation risk and patient preference must be proactively managed through counseling on expected gastrointestinal symptoms, gradual dose escalation strategies (per product labeling), and monitoring that integrates hepatic (transaminases, noninvasive steatosis assessment where available) and metabolic parameters (weight, HbA1c). Given the heterogeneity in weight response (I^2^ = 82%), clinicians should individualize expectations, reassess adherence and dose optimization, and recognize that smaller weight changes may still coincide with measurable liver fat reductions in some patients. When advanced fibrosis or cirrhosis is present, cautious interpretation is warranted, and referral to hepatology with consideration of trial enrollment remains appropriate given the persistent uncertainty around fibrosis reversal in later-stage disease with GLP-1 monotherapy.

Several limitations should be considered when interpreting these findings. The meta-analyses were based on a limited number of studies for several outcomes, with many pooled analyses including only two to four trials, which may reduce the precision and generalizability of the estimates. These limitations, together with risk-of-bias concerns and imprecision for some outcomes, resulted in GRADE certainty ratings ranging from very low to moderate. The included studies also varied in design, population, comparator, treatment duration, and outcome assessment methods, including MRI-PDFF, liver biopsy, and other imaging- or biomarker-based approaches. In addition, the presence of a single-arm study and multi-arm trials limits direct comparability across the evidence base. Treatment durations were generally short to moderate, and some studies included concomitant or combination therapies, making it difficult to attribute all observed effects solely to GLP-1 receptor agonist therapy. The substantial heterogeneity observed for body weight reduction (I^2^ = 82%) further suggests that pooled estimates may reflect important between-study differences in regimen intensity, baseline metabolic status, and comparator conditions.

## 5. Conclusion

This systematic review and meta-analysis suggest that GLP-1 receptor agonist-based therapy may improve hepatic steatosis and metabolic and biochemical parameters in patients with MASLD and MASH. Significant reductions were observed in liver fat content, body weight, ALT, AST, and HbA1c; however, these findings should be interpreted cautiously given the limited number of included studies and the small number of studies contributing to several pooled outcomes. GLP-1 receptor agonist therapy was associated with a higher frequency of gastrointestinal adverse events, particularly nausea and vomiting, while serious adverse events were not significantly increased. The current evidence primarily supports improvements in steatotic, biochemical, and metabolic surrogate outcomes rather than definitive histological or disease-modifying benefits. Evidence regarding MASH resolution, fibrosis regression, and long-term liver-related clinical outcomes remains limited. Larger, adequately powered randomized controlled trials with longer follow-up and standardized histological or validated non fibrosis endpoints are needed to determine whether these short- to medium-term improvements translate into sustained antifibrotic effects and meaningful long-term liver-related clinical benefits.

## Author contributions

**Supervision:** Mohammed Alfawaz.

**Validation:** Mohammed Alfawaz, Rahaf Alamri, Mohammed Abuladel, Rayan Alshehri.

**Writing – review & editing:** Mohammed Alfawaz, Yara Alorfi, Rahaf Alamri.

**Conceptualization:** Dareen Bajamaan.

**Methodology:** Dareen Bajamaan, Lama Alghamdi, Saud Khared.

**Investigation:** Lama Alghamdi, Nada Ameen, Rayan Alshehri, Shaima Altowairqi.

**Visualization:** Lama Alghamdi, Mohammed Abuladel.

**Writing – original draft:** Yara Alorfi, Saud Khared, Nada Ameen.

**Formal analysis:** Nada Ameen, Rahaf Alamri, Shaima Altowairqi.

**Resources:** Rayan Alshehri.
